# The anesthetic agent sevoflurane attenuates pulmonary acute lung injury by modulating apoptotic pathways

**DOI:** 10.1590/1414-431X20165747

**Published:** 2017-02-20

**Authors:** L. Wang, Y. Ye, H.B. Su, J.P. Yang

**Affiliations:** 1 Department of Anesthesiology, The First Affiliated Hospital of Soochow University, Suzhou, China; 2 Department of Anesthesiology, Suzhou Municipal Hospital-East, Suzhou, China; 3 Cam-Su Genomic Resource Center, Soochow University, Suzhou, China

**Keywords:** Sevofluran, Apoptosis, Pulmonary acute lung injury, Caspase-3, Bcl-2

## Abstract

The objective of this study was to evaluate lung protection by the volatile anesthetic sevoflurane (SEVO), which inhibits apoptosis. Male Sprague-Dawley rats (250–280 g; n=18) were randomly divided into three groups. The LPS group received 5 mg/kg endotoxin (lipopolysaccharide), which induced acute lung injury (ALI). The control (CTRL) group received normal saline and the SEVO group received sevoflurane (2.5%) for 30 min after ALI was induced by 5 mg/kg LPS. Samples were collected for analysis 12 h after LPS. Lung injury was assessed by pathological observations and tissue wet to dry weight (W/D) ratios. Apoptotic index (AI) was determined by terminal deoxynucleotidyl transferase dUTP nick-end labeling (TUNEL) assay and electron microscopy. Caspase-3 and cleaved-caspase-3 protein levels were determined by immunocytochemistry and western blotting, respectively. Bcl-xl levels were measured by western blotting and Bcl-2 levels by quantitative real-time polymerase chain reaction and western blotting. In the LPS group, W/D ratios, AI values, caspase-3 and cleaved-caspase-3 levels were significantly higher than in the CTRL group and lung injury was more severe. In the SEVO group, W/D ratios, AI, caspase-3 and cleaved-caspase-3 were lower than in the LPS group. Bcl-2 and Bcl-xl expression were higher than in the LPS group and lung injury was attenuated. Sevoflurane inhalation protected the lungs from injury by regulating caspase-3 activation and Bcl-xl and Bcl-2 expression to inhibit excessive cell apoptosis, and such apoptosis might be important in the pathogenesis of LPS-induced ALI.

## Introduction

Acute lung injury (ALI) is a highly lethal inflammatory lung disorder. During the onset of ALI, many types of damaging factors promote apoptosis of pulmonary vascular endothelial cells and alveolar epithelial cells, increasing lung tissue damage and contributing to the ALI inflammatory response (1). Though the exact mechanisms leading to pulmonary cell death are unknown, it is likely that apoptosis plays an important pathogenic role (2). ALI and acute respiratory distress syndrome (ARDS) can be of either pulmonary (direct) or extrapulmonary (indirect) origin (3) and, based on available evidence, direct and indirect ALI are truly different processes (4–8). While there is substantial overlap, studies estimate that approximately 55% of ARDS is caused by direct, rather than indirect, lung injury (9). Because many supportive therapies for ARDS fail (10,11), effective preventive strategies are needed. It was reported that sevoflurane postconditioning reduced endotoxin (lipopolysaccharide, LPS)-induced ALI, protecting lung function (12). Post-processing of inhalation anesthetics is a potential method for protecting the viscera from reperfusion injuries (13). Preconditioning with volatile anesthetics was shown to decrease apoptotic cell numbers and apoptosis regulatory protein levels both *in vivo* and *in vitro* (14,15). However, effects of sevoflurane preconditioning on apoptosis, during its protection of direct lung injury, are poorly understood. The aim of our study was to investigate effects of sevoflurane on LPS-induced ALI in rats and the possible mechanisms involved.

## Material and Methods

### Experimental animals and grouping

The Institutional Animal Care and Use Committee of Soochow University approved all experimental protocols. A total of 18 healthy male adult Sprague-Dawley rats, weighing 250-280 g, were maintained in the Soochow University experimental animal center. They were randomly divided into three groups: control (CTRL) group, LPS group and sevoflurane (SEVO) group. Pentobarbital (40 mg/kg) was injected endotracheally into each animal. After rats were anesthetized, the limbs were affixed to a board. Their necks were shaved, a medial 1 cm incision was made, and the trachea was exposed. The trachea was punctured with a 1 mL syringe with a 25-Ga needle and LPS or normal saline was slowly injected into the trachea over 30 s. After a bubble appeared, the incision was sutured. Rats in the SEVO group, which had also received LPS, were administered inhaled SEVO for 30 min. All rats were then returned to their cages and given food and water *ad libitum* for 12 h. They were then sacrificed by carotid arterial bleeding.

### Hematoxylin and eosin staining

The right lung inferior lobes were fixed with 10% formalin, embedded in paraffin, cut into 4-μm thick sections with a Leica RM2235 rotary microtome (Leica Biosystem, Germany) and mounted onto slides. After deparaffinization and hydration of the sections, they were stained with hematoxylin and eosin. Light microscopic examination was performed with a Nikon E400 microscope (Nikon Instrument Group, Japan).

### Transmission electron microscopy

Rat lung tissues were minced into small pieces and fixed in 2.5% glutaraldehyde in 0.1 M sodium cacodylate buffer for 4 h. Tissues were post-fixed in 1% osmium tetroxide in 1% K_4_Fe(CH)_6_, dehydrated through graded concentrations of ethanol and propylene oxide, embedded in Epon812 and then sectioned with an ultramicrotome. Longitudinal sections were placed onto copper grids, which were stained with uranyl acetate and lead nitrate and visualized with an H-600 electron microscope (Hitachi Limited, Japan).

### Lung wet weight to dry weight (W/D) ratios

After each rat was euthanized, the chest cavity was opened and the right middle lobe excised. Lung samples were rinsed in phosphate-buffered saline, blotted and weighed (wet weight). Subsequently, lung tissue samples were dried in an oven at 80°C for 48 h and again weighed (dry weight). The W/D ratio was then calculated.

### TUNEL assay

TUNEL assays were performed using an apoptosis detection kit (Roche Applied Science, Germany). Under the light microscope, TUNEL-positive cells had brown-stained nuclei. Five visual fields were selected randomly from each sample and at least 100 cells per field were counted at 200× magnification. The apoptotic index was calculated (apoptotic cells/total cells×100%) from a total of 25 fields per sample.

### Immunohistochemistry

A streptavidin-peroxidase immunohistochemical method was used to detect total caspase-3 protein. Tissue sections were deparaffinized and treated with 3% H_2_O_2_ for 5-10 min to inhibit endogenous peroxidase. After blocking with goat serum (BSA) for 30 min, rabbit anti-caspase-3 (1:500; Boster, China) primary antibody was added for overnight incubation at 4°C. Sections were then incubated with secondary antibody (1:700, Boster) at 37°C for 30 min. The stain was developed with diaminobenzidine and lightly counterstained with hematoxylin. Slides were then dehydrated and mounted under coverslips. Integrated optical density was determined with the Image-Pro Plus 7.0 software (Media Cybernetics, USA) at 400× magnification.

### Western blot analysis

The left lung inferior lobes were harvested 12 h after LPS instillation and homogenized in lysis buffer. The homogenate was incubated at 4°C for 30 min and then centrifuged (16,000 *g*) for 10 min at 4°C. Total protein content in each supernatant was measured by the BCA protein assay. Subsequently, samples containing an equal amount of protein, were separated on 15% SDS-PAGE gels and then transferred to a polyvinylidene difluoride membrane (Beyotime, China). After blocking with 5% fat-free milk, membranes were incubated with primary antibodies (CST, USA) against Bcl-2, Bcl-xl, cleaved-caspase-3 or β-actin overnight at 4°C. After washing three times with TBS-T, membranes were incubated at room temperature for 1 h in the presence of the secondary antibody (CST). Protein bands were visualized using the ECL system (Kodak system EDAS120, Japan).

### Quantitative real-time polymerase chain reaction (PCR)

Total RNA was isolated from left lung tissue samples using the Trizol reagent kit according to manufacturer instructions. Reverse transcription was performed at 40°C for 45 min followed by incubation at 95°C for 5 min. Quantitative real-time PCR was performed using SYBR Green on an Exicycler™ 96 real-time quantitative thermal block. The PCR primer sequences were designed according to the gene sequences reported in GenBank and were chemically synthesized: *Bcl-2* (upstream: 5′-ATCCCAGCCTCCGTTATCCT-3′, downstream: 5′-ATCCCAGCCTCCGTTATCCT-3′). The housekeeping gene β-actin was used for normalization. The ratios of the emissions incorporated into the PCR products of the tested gene to the signals for β-actin products were calculated to evaluate relative changes in mRNA expression levels. The relative amount of mRNA was counted by the 2−ΔΔCt method.

### Statistical analysis

Data are reported as means±SD. For experiments with more than 2 groups, one-way ANOVA was performed. P<0.05 was considered to be statistically significant.

## Results

### Sevoflurane attenuated pathological lung histological changes in LPS-induced pulmonary ALI

**Figure 1:**
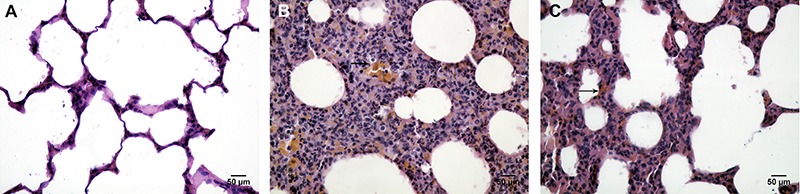
Lung histology detected by hematoxylin and eosin staining. *A*, Lungs of control animals had a normal appearance. B, After rats received lipopolysaccharide (5 mg/kg, LPS group), edema was severe, with increased red blood cells in the alveolar septum and inflammatory cells exiting blood vessels. *C*, At 30 min after inhalation of sevoflurane (2.5%; SEVO group), edema was significantly lower than in the LPS group, with fewer red blood cells in the alveolar spaces and far fewer inflammatory cells (magnification, 400×).

Pathological observations by light microscopy indicated no significant pathological changes in the CTRL group except for mild inflammation and capillary dilatation in some areas of the lungs (Figure 1A). In contrast, rats in the LPS group showed serious hyperemia and hemorrhage in the lung tissues, thickening and exudation of the alveolar walls and marked red blood cell and inflammatory cell infiltration in the alveolar spaces (Figure 1B). These pulmonary histopathological changes were attenuated significantly in the SEVO group, compared with the LPS group (Figure 1C).

### Changes in W/D ratios

**Figure 2:**
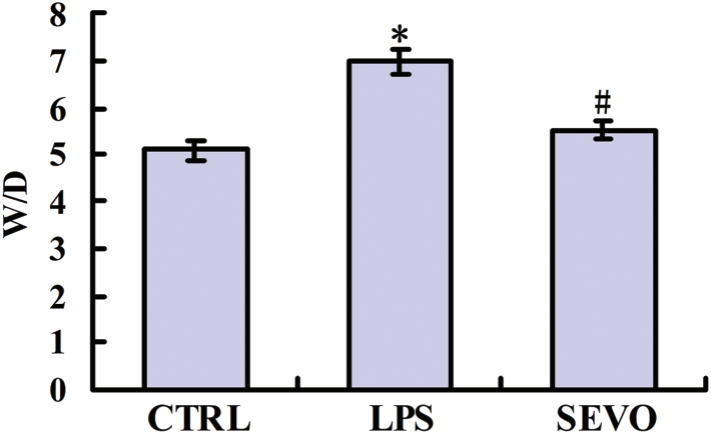
Lung wet to dry weight (W/D) ratios. The LPS group received 5 mg/kg lipopolysaccharide, which induced acute lung injury (ALI). The control (CTRL) group received normal saline and the SEVO group received sevoflurane (2.5%) for 30 min after ALI was induced. Data are reported as means±SD (n=6 per group). *P<0.01 *vs* CTRL group; ^#^P<0.01 *vs* LPS group (ANOVA).

The W/D ratios were significantly higher in the LPS group, compared with in the CTRL and sevoflurane groups (Figure 2).

### Lung tissue morphological changes

**Figure 3:**
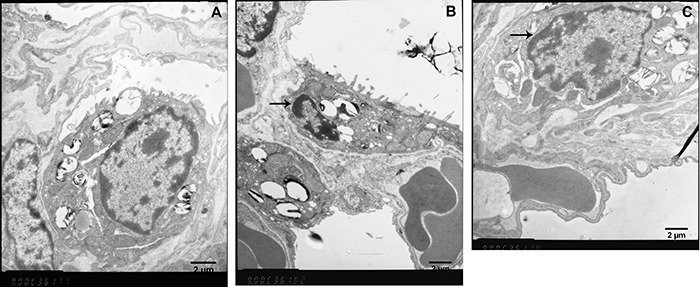
Transmission electron microscopy images of transverse sections of lung tissue. *A*, Air-blood barrier and endothelial structure had a normal appearance in the CTRL control group (saline). *B*, In the LPS group (5 mg/kg lipopolysaccharide), type II epithelial microvilli were absent or shortened, lamellar bodies were empty and chromatin was visible at the periphery of nuclei. *C*, There was no detectable apoptosis in the sevoflurane (2.5%) group (magnification, 8000×).

In the CTRL group, the alveolar walls were intact with a clear edge, type II alveolar epithelial microvilli were well-organized and the number of lamellar bodies, the air-blood barrier and endothelial structure appeared normal (Figure 3A). In contrast, in the LPS group, the alveolar walls were thickened, there was substantial alveolar epithelial edema and the alveolar epithelial cells showed slight swelling and degeneration. In addition, type II epithelial microvilli were missing or shortened, lamellar bodies were empty, chromatin was found at the periphery of nuclei, vascular endothelial cells were swollen and there were more pinocytotic vesicles. The alveolar compartments in the LPS group were enlarged and their electron density was decreased. Red blood cells and other substances were evident in the alveolar cavity (Figure 3B). The alveolar structure of the SEVO group indicated that there was significantly less damage than in the LPS group (Figure 3C).

### Apoptosis index in the lung tissues

**Figure 4:**
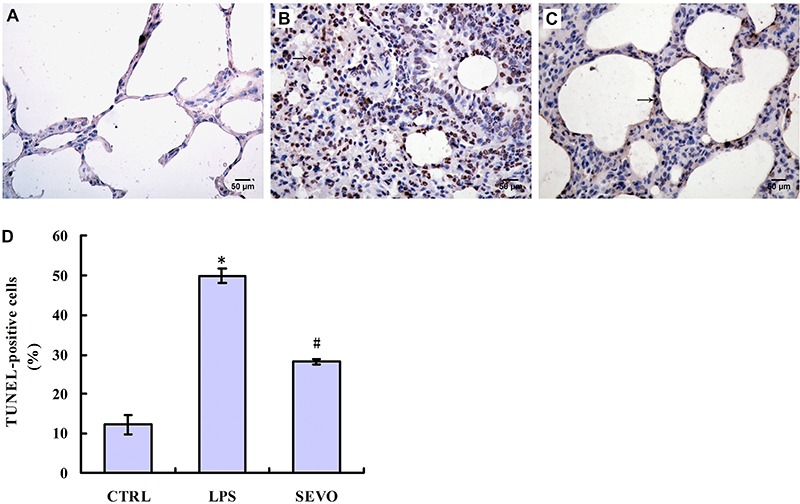
Apoptotic index (AI) in lung tissue samples. Data are reported as means±SD (n=6 per group). TUNEL assay results showing apoptosis (magnification, 400×) in the CTRL group (*A*), LPS group (*B*), and SEVO group (*C*). *D*, Relative AI values in the lung. CTRL: control group injected intratracheally with normal saline; LPS: injected intratracheally with lipopolysaccharide; SEVO: injected with LPS plus inhaled sevoflurane for 30 min. *P<0.01 *vs* CTRL group, ^#^P<0.01 *vs* LPS group (ANOVA).

In the LPS group, the AI increased significantly in the lung tissue, compared with the CTRL group. However, the percentage of apoptotic cells was significantly lower in the SEVO group than in the LPS group (Figure 4).

**Figure 5:**
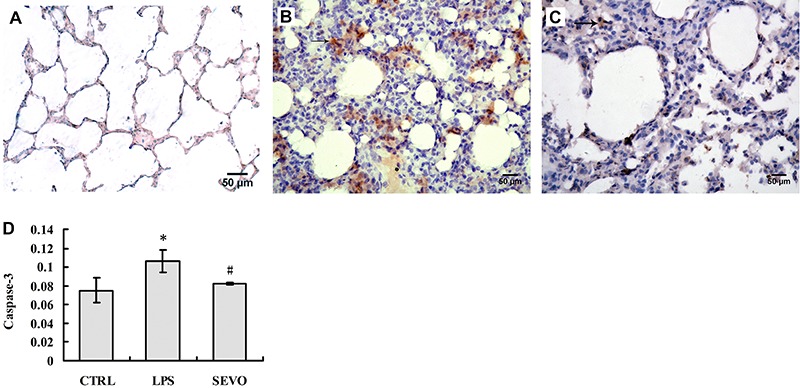
Caspase-3 levels in lung tissue samples. Immunohistochemical staining for caspase-3 (magnification, 400×) in the CTRL (*A*), LPS (*B*) and SEVO groups (*C*). *D*, Caspase-3 staining quantitation based on integrated optical densities in the lung tissues. Data are reported as means±SD (n=6 per group). CTRL: control group injected intratracheally with normal saline; LPS: injected intratracheally with lipopolysaccharide; SEVO: injected with LPS plus inhaled sevoflurane for 30 min. *P<0.01 *vs* CTRL; ^#^P<0.01 *vs* LPS (ANOVA).

Positive immunohistochemical brown staining for caspase-3 was specifically localized to the alveolar and bronchial epithelium. The number of cells expressing caspase-3 was significantly higher than controls in the lungs of rats subjected to LPS-induced ALI and were significantly decreased in rats also receiving sevoflurane treatment (Figure 5).

**Figure 6:**
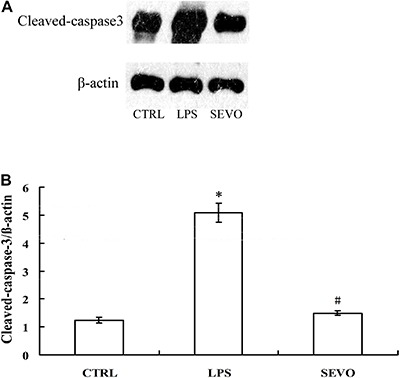
*A*, Western blotting for cleaved-caspase-3 in the lung tissues. *B*, Relative levels of cleaved-caspase-3. CTRL: control group injected intratracheally with normal saline; LPS: injected intratracheally with lipopolysaccharide; SEVO: injected with LPS plus inhaled sevoflurane for 30 min. Data are reported as means±SD (n=6 per group). *P<0.01 *vs* CTRL; ^#^P<0.01 *vs* LPS (ANOVA).

The lung levels of cleaved-caspase-3 determined by western blotting were significantly elevated compared with controls in LPS-induced ALI and were significantly decreased with sevoflurane treatment (Figure 6A and B).

### Regulation of Bcl-2 and Bcl-xl expression

**Figure 7:**
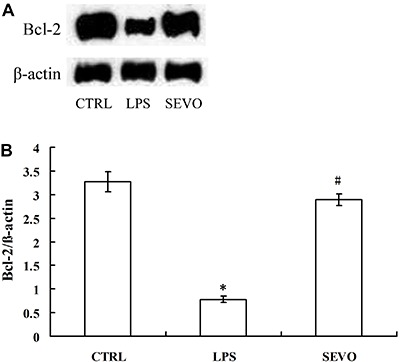
*A*, Western blot for Bcl-2 in the lung tissues. *B*, Relative levels of Bcl-2 protein expression. CTRL: control group injected intratracheally with normal saline; LPS: injected intratracheally with lipopolysaccharide; SEVO: injected with LPS plus inhaled sevoflurane for 30 min. Data are reported as means±SD (n=6 per group). *P<0.01 *vs* CTRL; ^#^P<0.01 *vs* LPS (ANOVA).

**Figure 8:**
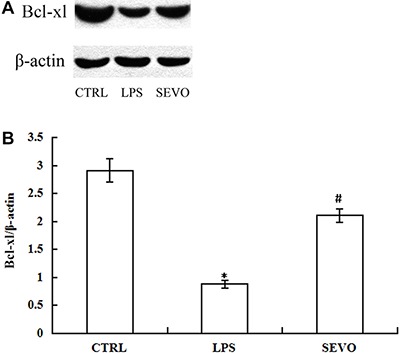
*A*, Western blot for Bcl-xl in lung tissue samples. *B*, Relative expression of Bcl-xl. CTRL: control group injected intratracheally with normal saline; LPS: injected intratracheally with lipopolysaccharide; SEVO: injected with LPS plus inhaled sevoflurane for 30 min. Data are reported as means±SD (n=6 per group). *P<0.01 *vs* CTRL; ^#^P<0.01 *vs* LPS (ANOVA).

Compared with the CTRL group, the levels of Bcl-2 and Bcl-xl in the LPS group were decreased. Compared with the LPS group, the ratios of Bcl-2 to Bcl-xl were higher in the SEVO group (Figures 7 and 8).

**Figure 9:**
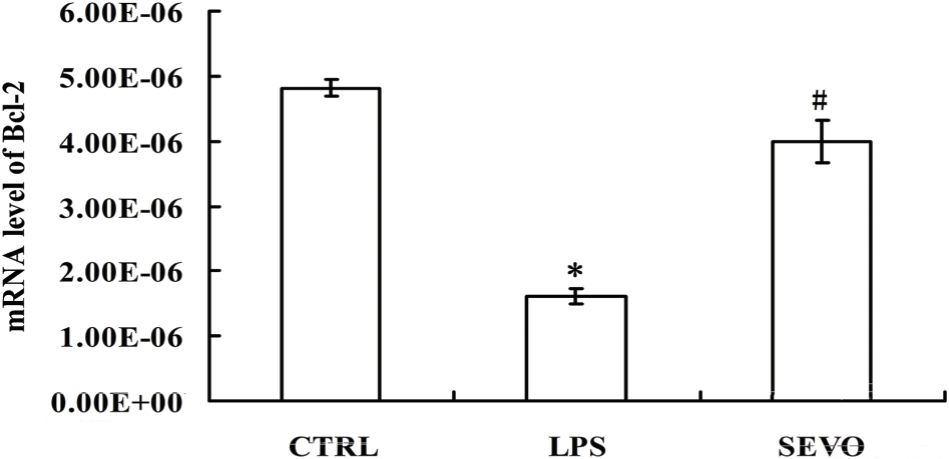
Relative levels of Bcl-2 in lung tissue samples assessed by real-time PCR. CTRL: control group injected intratracheally with normal saline; LPS: injected intratracheally with lipopolysaccharide; SEVO: injected with LPS plus inhaled sevoflurane for 30 min. Data are reported as means±SD (n=6 per group). *P<0.01 *vs* CTRL; ^#^P<0.01 *vs* LPS (ANOVA).

Bcl-2 mRNA was significantly lower in the LPS group compared with the CTRL group (Figure 9). In the SEVO group, Bcl-2 mRNA was higher than in the LPS group.

## Discussion

Although ARDS has been studied for nearly 40 years, its mortality rate remains as high as 30% (16). Pneumonia and aspiration of gastric contents account for most cases of direct lung injury while sepsis is the major cause of indirect lung injury (17,18). The most commonly used experimental models of direct lung injury are those using intratracheal LPS administration, mechanical ventilation or acid aspiration (19). Unfortunately, these simulate events that are often encountered in clinical anesthesia, so we chose a direct lung injury model for our study. We focused on examining regulation of apoptosis in a model of LPS-induced ALI.

Apoptosis is a mechanism for removing unwanted cells, thus limiting inflammation and tissue injury (20). Cytokines may inhibit inflammatory cell apoptosis, prolong inflammatory reactions and promote apoptosis of vascular endothelial cells and alveolar epithelial cells. We found an exudate 12 h after LPS instillation containing a large number of inflammatory cells, accompanied by alveolar and bronchial epithelial cell apoptosis. Ultimately, the number of inflammatory and apoptotic cells peaked at 12 h in our model. In inflammatory lung diseases, although molecular mechanisms of signaling are not completely understood, apoptosis of both lung epithelial cells and macrophages is proinflammatory and some results indicate that apoptosis generates inflammation in the lung, and suggest that caspases are promising targets for drug development. Blockade of Fas-FasL interactions either prevents or attenuates pulmonary inflammation (21).

Sevoflurane is an inhalation anesthetic commonly used in the clinic. When used, the bronchi and pulmonary alveoli are directly exposed to this agent. Previous studies confirmed the anti-apoptotic effects of sevoflurane on ischemia-reperfusion injury. In recent reports, sevoflurane pretreatment effectively attenuated lung injury by inhibition of neutrophil accumulation and alteration of the surfactant composition, attenuating the inflammatory response (22,23). When lung damage in the LPS group was the most severe, in the SEVO group was significantly less severe, indicated by apoptotic index and pathological morphology. As a potential mechanism, sevoflurane inhibited the abnormal increase in apoptosis of lung cells caused by LPS, so that the inflammatory response of lung tissue was decreased, leading to a lower degree of lung injury.

The anti-apoptotic effect of sevoflurane has been well reported. However, the anti-apoptotic mechanism of sevoflurane in primary lung injury has been less studied. To date, two major apoptotic pathways, intrinsic and extrinsic, have been described in mammalian cells. Both pathways converge at the level of caspase-3 activation (24,25). Some related research showed that enhanced caspase-3 activation was seen at 8 h. In our study, we observed an increased apoptosis rate 12 h after LPS. At this time-point, levels of cleaved-caspase-3 were significantly lower in the SEVO, compared with LPS group. Cell death is believed to be inevitable after cleavage of terminal caspases, with the most important being caspase-3. We propose, therefore, that sevoflurane can inhibit apoptosis by decreasing levels of caspase-3 and cleaved-caspase-3, blocking apoptosis at its final stage.

The Bcl-2 protein family is believed to determine the life or death status of cells. Members of this family include both pro-apoptotic and anti-apoptotic proteins (26), with Bcl-xl protein as the most well-known (27). Bcl-xl was shown to inhibit apoptosis by participating in a variety of protein–protein interactions. Husain et al. (28) reported that ALI was regulated by Bcl-2. In another report, sevoflurane anesthesia not only improved oxygenation and ameliorated inflammation, but also altered microRNA expression in a rat ALI model (29). Expression of the anti-apoptotic proteins Bcl-2 and Bcl-xl was increased by sevoflurane, while that of apoptotic proteins Bax and Bak was decreased, thus regulating hepatic apoptosis (30). In our study, the SEVO group had decreased number of TUNEL-positive cells in the lungs, compared with in the LPS group, as well as significantly higher levels of the early apoptotic signaling mediators, Bcl-xl and Bcl-2. Bcl-2 mRNA was similarly increased in the SEVO group to levels equivalent to those in the CTRL group. In mammals, Bcl-xl and Bcl-2 were shown to inhibit apoptosis by interfering with the activity of caspase-3 (31). Therefore, we believe that sevoflurane pretreatment can lead to maintenance of a relatively high concentration of the anti-apoptotic proteins Bcl-xl and Bcl-2, blocking caspase-3-mediated apoptosis.

In conclusion, our study demonstrated the following: 1) sevoflurane preconditioning inhibited development of apoptosis in LPS-induced ALI; 2) sevoflurane-mediated anti-apoptotic activity was associated with decreased levels of caspase-3 and cleaved-caspase-3 and upregulation of Bcl-xl and Bcl-2; and 3) sevoflurane preconditioning is a potential therapeutic strategy to prevent lung injury resulting from aspiration events. Further studies are needed to confirm whether epithelial cells are protected by sevoflurane preconditioning.
